# Cultivation technology development of *Rhodothermus marinus* DSM 16675

**DOI:** 10.1007/s00792-019-01129-0

**Published:** 2019-09-14

**Authors:** Emanuel Y. C. Ron, Roya R. R. Sardari, Richard Anthony, Ed W. J. van Niel, Gudmundur O. Hreggvidsson, Eva Nordberg-Karlsson

**Affiliations:** 1 Division of Biotechnology, Department of Chemistry, Lund University, Naturvetarvägen 14, 22100 Lund, Sweden; 2 Division of Applied Microbiology, Department of Chemistry, Lund University, Naturvetarvägen 14, 22100 Lund, Sweden; 3 Matis Ohf, Vinlandsleid 12, 113 Reykjavik, Iceland

**Keywords:** *Rhodothermus marinus*, Carotenoid, Exopolysaccharide, Sequential batch cultivation, Bioreactor

## Abstract

This work presents an evaluation of batch, fed-batch, and sequential batch cultivation techniques for production of *R. marinus* DSM 16675 and its exopolysaccharides (EPSs) and carotenoids in a bioreactor, using lysogeny broth (LB) and marine broth (MB), respectively, in both cases supplemented with 10 g/L maltose. Batch cultivation using LB supplemented with maltose (LB_malt_) resulted in higher cell density (OD_620_ = 6.6) than use of MB_malt_ (OD_620_ = 1.7). Sequential batch cultivation increased the cell density threefold (OD_620_ = 20) in LB_malt_ and eightfold (OD_620_ = 14) in MB_malt_. In both single and sequential batches, the production of carotenoids and EPSs using LB_malt_ was detected in the exponential phase and stationary phase, respectively, while in MB_malt_ formation of both products was detectable in both the exponential and stationary phases of the culture. Heteropolymeric EPSs were produced with an overall volumetric productivity (*Q*_E_) of 0.67 (mg/L h) in MB_malt_ and the polymer contained xylose. In LB, *Q*_E_ was lower (0.1 mg/L h) and xylose could not be detected in the composition of the produced EPSs. In conclusion, this study showed the importance of a process design and medium source for production of *R. marinus* DSM 16675 and its metabolites.

**Electronic supplementary material:**

The online version of this article (10.1007/s00792-019-01129-0) contains supplementary material, which is available to authorized users.

## Introduction

*Rhodothermus marinus* is a thermophilic aerobic heterotrophic bacterium that was first isolated from a submarine hot spring in Isafjardardjup in Iceland (Alfredsson et al. 1988). At first, *R. marinus* was primarily of interest because of its thermostable enzymes (Crennell et al. 2002; Hobel et al. 2005; Karlsson et al. 2002; Nordberg Karlsson et al. 1997; Spilliaert et al. 1994). More recently, strain *R. marinus* DSM 16675 (previously published as strain PRI493 and MAT493) has been a target for research due to the developed genetic tools, enabling genetic engineering for the first time in the genus (Bjornsdottir et al. 2011). Moreover, both wild-type and engineered strains of *R. marinus* have been used to study two different groups of natural products, i.e., exopolysaccharides (EPSs) (Sardari et al. 2017) and carotenoids (Ron et al. 2018). Marine exopolysaccharides have unique properties which enable them to have potential applications in different industries such as food and pharmaceutical industries (Nwodo et al. 2012). Likewise, microbial carotenoids have a wide range of applications especially in food and medical industries (Kaiser et al. 2007).

During the study of the natural products of *R. marinus*, it became apparent that there is a lack of knowledge on cultivation technology for reaching high cell density of *R. marinus,* which is a necessity to increase the yield and productivity of the products. Only a handful of studies have published growth data on *R. marinus*. In most cases, these studies have focused on increasing the production of specific carbohydrate-converting enzymes, including xylanolytic enzymes (Dahlberg et al. 1993), β-mannanase (Gomes and Steiner 1998), α-l-arabinofuranosidase (Gomes et al. 2000), α-galactosidase (Blücher et al. 2000), amylase and pullulanase (Gomes et al. 2003), in all cases using batch cultivation at low cell densities. Recently, ^13^C flux was also reported for the type strain of *R. marinus* (DSM4252), but also in this case very low cell densities (OD < 2) were reached (Cordova et al. 2017). The aim of this study was to evaluate the different cultivation techniques and media to obtain higher cell densities of *R. marinus* DSM 16675 and to investigate the metabolite formation kinetics.

## Materials and methods

### Chemicals

Yeast extract and tryptone were obtained from Duchefa Biochemie, Marine Broth 2216-Difco from Becton Dickinson and NaOH (50%) from Merck. All other chemicals were from Sigma-Aldrich.

### Microorganism and inoculum preparation

*Rhodothermus marinus* DSM 16675 (also known as MAT/PRI 493) was received from Matís ohf, Reykjavík, Iceland. The cells were revived on solid agar plates with modified *Thermus* 162 medium with 1% NaCl at 65 °C for 24–48 h according to DSMZ specifications (Degryse et al. 1978). Colonies were transferred to 5 mL of liquid medium [Lysogeny broth (LB) including 10 g/L tryptone, 10 g/L NaCl, and 5 g/L yeast extract, or marine broth (MB) (according to DSMZ specifications)] in 50 mL falcon tubes for incubation (Ecotron, Infors) at 65 °C and 200 rpm for 24 h. After the overnight incubation, the cells were transferred into 50 mL of liquid medium in a 500 mL baffled shake flask and incubated for 8 h (OD_620nm_ ≈ 5 for LB and OD_620nm_ ≈ 1.5 for MB) before inoculating the bioreactor (Multifors 2, Infors). All cultivations were done using 10% (v/v) inoculum grown in liquid medium.

### Culture media preparation

LB supplemented with 10 g/L glucose (LB_glu_) or maltose (LB_malt_), and MB supplemented with maltose (MB_malt_) were used in the different cultivations. The liquid medium was prepared and autoclaved at 121 °C for 20 min. A stock solution of sugar with a concentration of 100 g/L was prepared and filter sterilized and added to the culture medium to reach the final concentration of 10 g/L.

### Cultivation conditions in stirred tank bioreactor

#### Batch cultivation

Batch cultivation was performed in a 1 L stirred tank bioreactor (Multifors 2, Infors) with a working volume of 0.5 L. The temperature was set at 65 °C and the pH of the medium in the bioreactor was maintained at 7.0 by addition of 1 M NaOH. The bioreactor was aerated with filter-sterilized air at a constant rate of 0.5 VVM (air volume/culture volume/min). The initial agitation speed was 200 rpm and cascaded with a dissolved oxygen tension (DOT) probe. The DOT was maintained at setpoint of 40% by the DOT probe (instructing the bioreactor engine to increase the agitation). The percentage of CO_2_ in the outgas was measured online by a gas analyzer (Gas analyzer, Infors). The antifoam (Y-30) was used during the cultivation to suppress foam formation. The antifoam was diluted 50 times before use since the concentrated antifoam was toxic for cells. Samples were taken during 24 h of cultivation and the OD of each sample was measured at 620 nm.

#### Fed-batch cultivation

The fed-batch cultivation of *R. marinus* DSM 16675 was started in normal batch mode. After inoculation, the batch was started with 350 ml of the LB medium supplemented with 5 g/L maltose and the OD was monitored every 2 h. When the growth rate decreased and the OD was almost constant, meaning the cells reached stationary phase, the fed-batch mode was initiated by addition of the feed solution. Stepwise feeding was used and a concentrated feed was prepared with equivalent ingredient proportions (100 g/L maltose, 100 g/L yeast extract, 200 g/L tryptone, and 10 g/L NaCl) and added to the bioreactor using a syringe. The salinity of the medium should remain constant during the fed-batch, hence the salt concentration was unchanged in the feed. The volume fed to the bioreactor was calculated based on the carbohydrate already consumed at the time of feeding, so that it would reconstitute the concentration of sugar. Samples were taken and the OD of each sample was measured at 620 nm.

#### Sequential batch cultivation with cell recycling

Two sequential batch cultivations of *R. marinus* DSM 16675 were carried out. The first one used LB_malt_ with a total of seven consecutive batches with cell recycling and the second used MB_malt_ in four consecutive batches. The first batch cultivation was inoculated with 50 ml of inoculum (10% v/v) to 450 ml of the medium in the bioreactor. The OD measurement was performed every 1 or 2 h for each batch and when the cells in each batch reached the stationary phase, the cultivation was terminated and the medium containing cells was transferred to 250 mL sterile centrifuge tubes and pelleted at 7000 rpm and 20 °C for 10 min. The supernatant was discarded while the pellet was dissolved in 500 ml of pre-heated (65 °C) fresh medium and transferred to the bioreactor. This process was repeated and the samples were taken until termination of the cultivation.

### Analytical methods

Samples were taken with regular intervals during the cultivation, and used for determining cell growth (spectrophotometrically as optical density (OD) at 620 nm), substrate consumption, organic acid determination, and spectrophotometric estimation of carotenoids.

#### EPSs and substrate analysis and quantification

Total produced EPSs were determined by quantifying their total monosaccharide content after acid hydrolysis. Samples from the cultivation were centrifuged and the crude EPSs were precipitated with four volumes of ethanol (99%) from the cell-free supernatant and lyophilized. The monosaccharides were analyzed after hydrolysis of lyophilized crude EPSs with sulfuric acid, neutralization with 0.1 M Ba (OH)_2_, and subsequently proper dilution of the samples (Sluiter et al. 2008). Substrate consumption and monosaccharide content of the produced exopolysaccharides (EPSs) were determined by high-performance anion-exchange chromatography (HPAEC) (Thermo Fisher Scientific, Waltham, USA) using a Dionex CarboPac PA-20 analytical column which was coupled to a Dionex CarboPac PA-20 guard column. Three pumps were used for three different eluents: Pump A (Milli-Q water), pump B (2 mM NaOH), and pump C (200 mM NaOH). Separation occurred during 23 min of running time using a mixture of A (62.5%) and B (37.5%) with an isocratic flow of 0.5 mL/min and after that the column was regenerated with C (100%) for 2 min at the same flow rate. Disaccharide analysis was carried out using a mixture of A (50%) and C (50%) with an isocratic flow of 0.5 mL/min and the running time was 20 min. The analytes were detected with an ED40 electro-chemical detector. Before injection in the Dionex system, the cell-free supernatant was filtered through a 0.2 µm polypropylene filter after proper dilution and analyzed for the remaining substrate in each sample.

The total volumetric productivity of produced EPSs (*Q*_E_) was calculated as the sum of *Q*_E_ in each batch, as follows:

$$Q_{{\text{E,total}}} = \frac{{\mathop \sum \nolimits_{i = 1}^{n} \Delta P_{i} }}{{t_{{{\text{total}}}} }}\quad {\text{with}}\,\,\Delta P_{i } = \left[ {{\text{EPSs}}} \right]_{{{\text{final}}}} {-} \, \left[ {{\text{EPSs}}} \right]_{{{\text{initial}}}} \,\,\,$$

The *n* and *t* indicate the number of batches and total time (h) of the sequential batch cultivation, respectively, and $\Delta P_{i}$ is the EPS formation during each single batch *i*. The *Q*_E_ of each batch was calculated as $\Delta P$ divided by the time of each batch.

#### Carotenoids analysis

The carotenoids were extracted from the *R. marinus* DSM 16675 strain during batch, fed-batch, and sequential batch cultivations. The extraction was performed according to the method described by Biehler et al. (Biehler et al. 2010). One milliliter of culture sample was pelleted down at 13,000 rpm for 5 min and the pellet was re-suspended in 1 mL of acetone (99.5%), shaken for 1 h on a rocking table at room temperature and then, separated from the supernatant by centrifugation at 13,000 rpm for 3 min. The absorbance of the cell-free supernatant was measured at 450 nm using a UV quartz cuvette (Hellma) in a UV/Visible spectrophotometer (Pharmacia biotech, Ultrospec 1000) and plotted against the cultivation time.

#### Organic acid analysis

Organic acid determination was performed using high-performance liquid chromatography (HPLC). After proper dilution, 1 mL of a cell-free sample was acidified with 20 µL of sulfuric acid (20% v/v), and filtered through 0.2 µm polypropylene filter, prior to analysis in the HPLC system (HPLC Ultimate-3000 RSLC, Dionex) connected to an IR detector (Shodex, RI-101). Separation of organic acid was done using the analytical column Aminex HPX-87H connected to a guard column (Biorad, Richmond, CA, USA). The temperature was set at 40 °C and the mobile phase consisted of 5 mM H_2_SO_4_ with a flow rate of 0.5 mL/min.

## Results and discussion

### Single batch cultivation of *R. marinus* DSM 16675

**Fig. 1:**
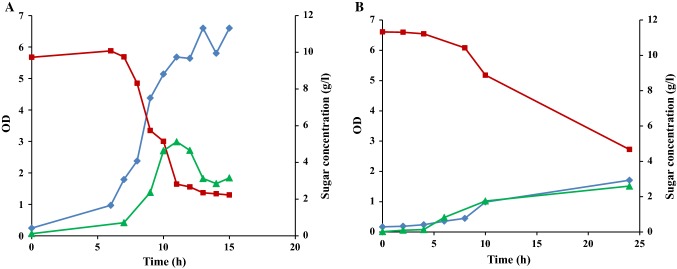
Growth profile of *R. marinus* DSM 16675 and sugar consumption during batch cultivation in **a** LB_malt_ and **b** MB_malt_. Symbols: (filled diamond) cell density represented by OD_620nm_, (filled square) concentration of maltose (g/L), (filled triangle) concentration of glucose (g/L)

A previous study, focusing on shake flask cultivations in marine broth (MB) supplemented with different mono- and disaccharides, showed that the highest growth of *R. marinus* DSM 16675 (0.75 g/L) was reached using supplementation with maltose (MB_malt_) (Sardari et al. 2017). Therefore, in this study, maltose was chosen as a supplementary carbon source and was added to cultivation of *R. marinus* DSM 16675, run in complex media (LB or MB) using a stirred tank bioreactor with controlled aeration and pH (Fig. 1). Growth of *R. marinus* DSM 16675 was initiated after a lag phase of 6 and 4 h in LB_malt_ and MB_malt_, respectively, followed by an exponential growth phase lasting until 10 h cultivation time, reaching a maximum specific growth rate (μ_max_) of 0.42 h^−1^ and 0.22 h^−1^ in LB_malt_ and MB_malt_, respectively. After 10 h, the cell culture entered the stationary phase, as monitored by cease of growth. Consumption of maltose in cultivations using LB_malt_ was detected between 6 and 10 h cultivation time at an exponential rate, which decreased significantly after 10 h. In the MB_malt_ cultivation, maltose consumption started after 4 h (indicating start of the exponential growth phase) with a fast consumption rate until 10 h, followed by a period with gradually decreasing maltose concentration until the end of cultivation. It should be noted that accumulation of glucose was seen in both media from the start of the exponential phase until the end of the cultivation, meaning that not all of the maltose was taken up and consumed, but rather enzymatically degraded to its monosaccharide constituents (Fig. 1). Moreover, the obtained glucose in LB medium was consumed for 2 h during the stationary phase without display of cell growth and thus might be used for cells’ maintenance (Navarro Llorens et al. 2010). The maximum OD_620_ in LB_malt_ was 6.6 after 13 h which was significantly higher than the OD observed in MB_malt_ where a maximum OD_620_ of 1.71 was observed after 24 h.

**Fig. 2:**
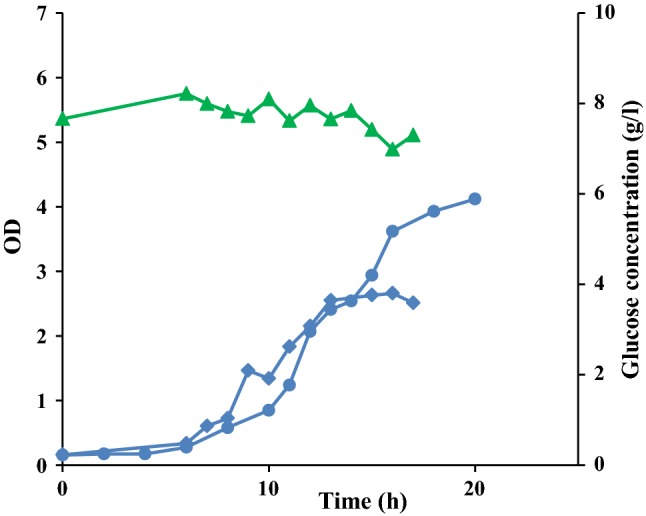
Comparison of growth profiles of *R. marinus* DSM 16675 in batch cultivations in LB and in LB supplemented with glucose in bioreactor. Symbols: (filled diamond) cell growth in LB_glu_, (filled circle) cell growth in LB medium, (filled triangle) concentration of glucose in the bioreactor cultivation

In the previous study (Sardari et al. 2017) with shake flask cultivations, it was shown that glucose supplementation in MB (MB_glu_) resulted in a slight increase in the cell growth of *R. marinus* DSM 16675 and EPSs production. Hence, cultivation of *R. marinus* DSM 16675 was performed to investigate the growth behavior using LB medium supplemented with 10 g/L glucose (LB_glu_) at controlled aeration and pH in the bioreactor (Fig. 2). A bioreactor cultivation with LB (without supplementation) was also run in parallel. The cultivation data displayed that glucose supplementation had no effect on the cell growth and only low (if any) uptake of glucose could be detected during the experiment. A maximum OD_620_ of 2.7 was reached after 16 h in the bioreactor cultivation using LB_glu_, which was less than that of the cultivation using LB with no additional carbon source (OD_620_ of 3.6 after 16 h).

### Product formation kinetics in single batch cultivations

**Fig. 3:**
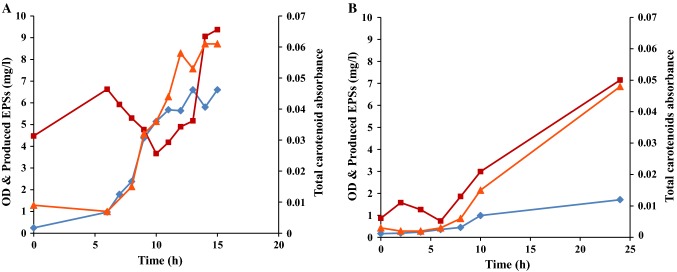
Cell growth and product formation profiles during batch cultivation of *R. marinus* DSM 16675 in **a** LB_malt_ and **b** MB_malt_. Symbols: (filled diamond) cell growth presented as OD_620nm_, (filled triangle) total carotenoids absorbance at 450 nm, and (filled square) EPSs concentration (mg/L)

*R. marinus* DSM 16675 has been shown to form two products of potential application interest: carotenoids (Ron et al. 2017) and exopolysaccharides (Sardari et al. 2017). To increase the understanding of the formation of these products, the relationship between cell growth and the production of carotenoids and EPSs in single batch cultivations in a pH-controlled bioreactor using LB_malt_ and MB_malt_ was studied (Fig. 3). It should be noted that LB and MB (except for the added maltose) contain very low concentrations of sugars, as previously reported by Sezonov et al. (Sezonov et al. 2007) which were precipitated and hydrolyzed together with the EPSs from the medium (Fig. 1 and 2, supplementary materials). The reduction in those sugars was observed between 6 and 10 h cultivation in LB_malt_ (Fig. 3a) and between 2 and 6 h in MB_malt_ (Fig. 3b).

**Fig. 4:**
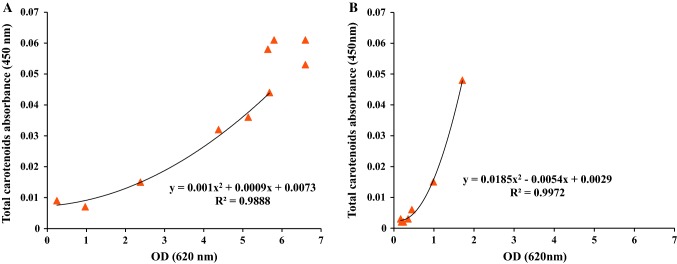
Changes in total carotenoids absorbance as a function of cell growth represented as OD_620nm_ in **a** LBmalt and **b** MBmalt

As seen in Fig. 3a, the batch cultivation in LB_malt_ produced carotenoids starting 6 h after inoculation and continued until the end of the exponential phase. The total produced carotenoids profile, represented by absorbance at 450 nm and a final absorbance of 0.061, was best fitted to a second-order polynomial equation using Excel software (Microsoft, 2010) in the exponential phase of the cultivation (Fig. 4a). On the other hand, the changes in EPSs concentration were more difficult to evaluate, with apparent formation only seen from the onset of the stationary phase reaching a final net formation of 5.18 mg/L at the end of cultivation.

In MB_malt_ a different production pattern of carotenoids and EPSs was observed. Figure 3b depicts that production of both total carotenoids and EPSs started at the beginning of the exponential phase and continued during the stationary phase for both products, reaching a total carotenoids final absorbance at 450 nm of 0.048 and a final EPSs formation of 6.28 mg/L. It can be speculated that the increase in EPSs formation during the stationary phase can be a result of quorum sensing activity that consequently stimulates formation of biofilm (Sengupta et al. 2018). In addition, cell lysis can contribute to biofilm development in which the EPSs are the main components (Bayles 2007; Flemming et al. 2007). Bayles (Bayles 2007) described that the DNA released from the cells during cell lysis at the stationary phase is a structural component of the biofilm matrix. However, in our case the rate of cell lysis seems to be low since the OD was not decreased at the stationary phase.

The total produced carotenoids profile, also, was fitted to a second-order polynomial equation for exponential phase and stationary phase of the batch cultivation (Fig. 4b).

The ratio of EPSs to cell growth (OD) was 1.41 and 4.18 for LB_malt_ and MB_malt_, respectively. This indicates a strong competition between cell growth and EPS production as previously described (Sardari et al 2017). In addition, analysis of the monosaccharide composition of the EPSs hydrolysate from the cultivations in both media was performed to quantify the total EPSs. It showed that the monosaccharides of the produced EPSs from LB_malt_ consisted of arabinose, galactose, glucose, and mannose. For the EPSs in the culture grown in MB_malt_ the same monosaccharides were found, but with the addition of xylose, which is in accordance with the previously published data on the EPSs composition from *R marinus* grown in this medium (Sardari et al. 2017) (Fig. 3, supplementary materials).

The cells produced carotenoids in both media, but interestingly, the production of carotenoids continued in the stationary phase when the cells were grown in MB_malt_ (Fig. 3b). This might be due to accumulation of tricarboxylic acid cycle metabolites with subsequent conversion to other products such as carotenoids (Henke et al. 2017; Tao et al. 2011).

Production of short chain fatty acids (SCFA) was investigated to explain the decrease in pH during the cultivation, and corresponding consumption of NaOH (1 M), approximately 3 mL in LB_malt_ and 0.5 mL in MB_malt_, during the batch cultivations of *R. marinus* DSM 16675 in both media. However, very little lactic acid, formic acid, and acetic acid were produced. The main reason for the drop in pH might instead be due to the reaction of the produced CO_2_ with water and formation of carbonic acid in the culture media (Das and Mangwani 2015).

As seen in Fig. 1, both maltose and glucose were left in the culture medium at the end of both cultivations, excluding that the carbon source is the limiting factor for growth. The highest cell densities were reached in the batch cultivations of *R. marinus* DSM 16675 using LB_malt_ as growth medium. Hence, LB_malt_ was selected as growth medium for fed-batch cultivation of *R. marinus* DSM 16675, aiming at further increasing cell densities, and solve the eventual nutrient limitations occurring towards the end of the batch cultivations.

### Fed-batch cultivation of ***R. marinus*** DSM 16675

**Fig. 5:**
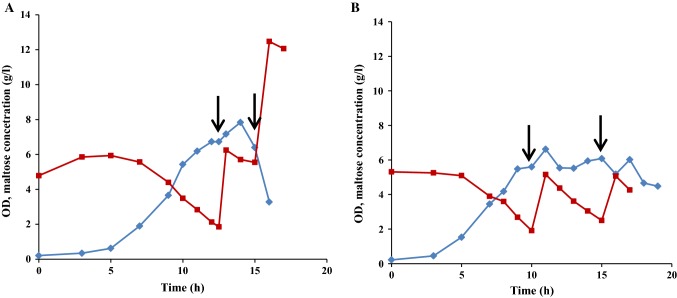
Growth profile of *R. marinus* DSM 16675 and sugar consumption during **a** fed-batch cultivation in LB_malt_ and **b** repeated fed-batch cultivation in LB_malt_. Symbols: (filled diamond) cell growth represented as OD_620nm_, and (filled square) maltose concentration (g/L). Feeding was made stepwise, as indicated by arrows

Fed-batch cultivation with stepwise feeding was applied for *R. marinus* DSM 16675 using LB medium supplemented with 5 g/L of maltose in the bioreactor (Fig. 5).

As seen in Fig. 5a, the cell growth reached the stationary phase after 12 h of cultivation at an OD of 6.73 after consumption of approximately 3 g/L maltose. The first feed, which consisted of 20 mL of feed solution, was added at 12.5 h of cultivation and growth resumed. The maximum OD reached was 7.84 after 14 h, which decreased to 6.4 after 1 h. At that point, the second feed was added at 15 h of cultivation, but growth was not recovered. Instead, the OD dropped unexpectedly. The maximum OD achieved in the fed-batch cultivation did not exceed the OD in the prior batch cultivations. This could theoretically be due to substrate inhibition, since the concentration of maltose reached 12.47 g/L after the second feed addition. However, repeated fed-batch cultivations with maltose concentrations never exceeding 5 g/L also reached the stationary phase at the same cell densities (Fig. 5b).

Other potential explanations for the termination of growth at 14 h could be either inhibition due to accumulation of growth-inhibiting metabolites, or quorum sensing. To distinguish between these alternatives, sequential batch cultivation with cell recycling was selected as a method to circumvent metabolites reaching growth-inhibiting levels.

#### Sequential batch cultivation of ***R. marinus*** DSM 16675 with cell recycling

**Fig. 6:**
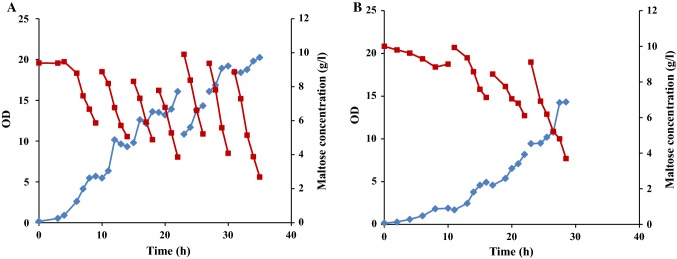
Growth profile of *R. marinus* DSM 16675 and sugar consumption during sequential batch cultivation using **a** LB_malt_ and **b** MB_malt_. Symbols: (filled diamond) cell growth represented as OD_620nm_ and (filled square) maltose concentration (g/L)

Since the fed-batch cultivations were not successful in increasing biomass concentration, sequential batch cultivation of *R. marinus* DSM 16675 with cell recycling in LB_malt_ and MB_malt_ was investigated (Fig. 6).

The first batch cultivation, of *R. marinus* DSM 16675, in the cultivation sequence in the respective medium was started with the addition of a 10% (v/v) inoculum followed by an exponential phase with a duration of 5 h (cultivation time 3–8 h), reaching the stationary phase after 9 h of cultivation, and a final OD_620nm_ of 5.68 and 1.82 in LB_malt_ and MB_malt_, respectively (Fig. 6). At that point, the cultivation was stopped and the next batch was prepared with fresh medium and inoculated with the recycled cells from the previous batch. Between batch 4 and batch 5, a drop in the starting cell density was observed (compared to the final cell density of the previous batch cycle). This must be due to loss of cells during handling between the cycles while removing the old medium and adding fresh medium. After seven cycles using LB_malt_, the maximum OD of 20.2 was reached after 35 h cultivation time, resulting in a threefold increase relative to that of the batch and fed-batch trials. To the best of our knowledge, the final OD value of 20.2 is the highest cell density achieved with the genus according to literature (Fig. 6a). Our results are comparable with the results obtained with other similar thermophilic bacteria that were cultivated to reach higher cell densities (Table 1). As can be seen, sequential batch with cell recycling is as effective as other fermentation techniques to produce high cell densities with thermophiles.

**Table 1:** Comparison of different techniques for high cell density cultivation of thermophiles

	Bacteria	Cultivation technique	Cultivation volume (L)	Cultivation time (h)	Cell mass productivity (mg L^−1^ h^−1^)	Cell density (CDW g/L)	References
Aerobic/facultative anaerobic	*Chelatococcus *sp. MW10	Cyclic batch	75	140	3.2	34	(Ibrahim and Steinbüchel 2010)
	*Saccharolobus shibatae*	Dialysis fed-batch	406	358	0.78	114	(Krahe et al. 1996)
	*Chelatococcus *sp. MW10	Cyclic fed-batch	35	265	12	115	(Ibrahim and Steinbüchel 2010)
	*Rhodothermus marinus* DSM 16675	Sequential batch with cell recycling	3.5	35	40	4.91^a^	This study
	*Thermus thermophilus*	Batch	2	15	100	3	(Demirtas et al. 2003)
	*Geobacillus LC300*	Fed-batch	1	10.4	650	6.75	(Cordova et al. 2015)
Anaerobic	*Thermoanaerobacter brockii*	Continuous with cell recycling	20	32	10	6.5	(Holst et al. 1997)

^a^Correlation between CDW and OD of *R. marinus* DSM 16675 was obtained as CDW = 0.2341 OD + 0.2371 (0.2 ≤ OD ≤ 6, *R* = 0.9735)

The maximum OD of 14.32 was reached over four cycles in the MB_malt_ medium after 30 h of cultivation, which is an eightfold increase relative to that of the batch trial (Fig. 6b).

The higher cell density in LB_malt_ medium can be due to the higher concentration of yeast extract in LB_malt_ (5 g/L) compared to that in MB_malt_ (1 g/L). Yeast extract generates high cell densities for Gram-negative bacteria as described by Gray (Gray et al. 2008). Also, LB_malt_ has twice as much of tryptone (casein peptone) (10 g/L) as peptone in MB_malt_ (5 g/L), which can explain the higher cell densities in LB_malt_ compared to MB_malt_ Another difference is the salt composition and concentration, which seems to slightly hinder growth of *R. marinus* DSM 16675 in MB_malt_. Marine broth emulates open seawater salt composition and has therefore a higher NaCl (2% (w/v)) concentration compared to LB (1% (w/v)).

In each cultivation cycle, the stationary phase was reached before depletion of maltose, which is in line with results from the prior batch cultivations, most likely due to depletion of other nutrients or accumulation of growth-inhibiting products.

The maximum specific growth rate (*μ*_max_) was 0.42 h^−1^ for the first batch cultivation in LB_malt_ and 0.26 h^−1^ for the first batch cultivation in MB_malt_, which is in accordance with the specific growth rates observed in the single batch cultivations (described in Sect. 3.1.1). However, the specific growth rate decreased gradually in the later batches until the end of cultivation. This shows that the limitation in growth in the fed-batch (described in Sect. 3.2) may have been caused by accumulation of growth-inhibiting metabolites, alleviated by the change of medium in the sequential batch. However, the reasons for the decreased growth rate, in the later cycles in the batch sequence remain unclear and may be influenced by quorum sensing. Reaching a high cell density in a batch culture is a sign for cells to predict severe competition for nutrients and subsequently lack of nutrients. Entry to the stationary phase is a main solution for bacteria to survive. Therefore, the quorum sensing system (Kaur et al. 2018; Montgomery et al. 2013) regulates the transition into stationary phase by quorum sensing signals which are produced mainly at high cell density (Lazazzera 2000).

#### Product formation kinetics in the sequential batches

**Fig. 7:**
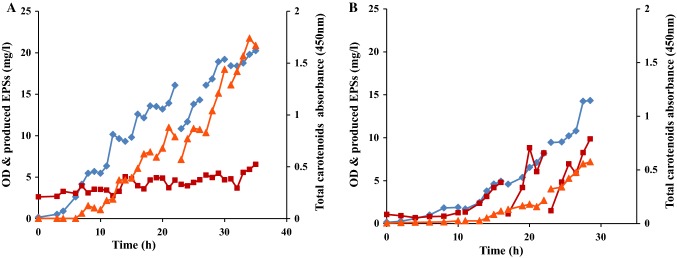
Cell growth and product formation profile during sequential batch cultivation of *R. marinus* DSM 16675 in **a** LB_malt_ and **b** MB_malt_. Symbols: (filled diamond) cell growth represented as OD_620nm_ (filled triangle) total carotenoids absorbance, and (filled square) EPSs concentration (mg/L)

The production of carotenoids and EPSs was evaluated in the sequential batch cultivations of *R. marinus* DSM 16675 in both LB_malt_ and MB_malt_ (Fig. 7).

**Fig. 8:**
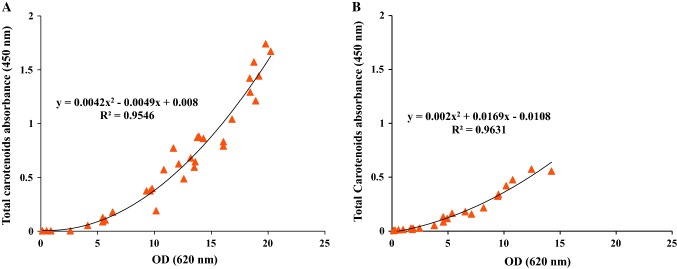
Changes in total carotenoids absorbance as a function of cell density represented as OD_620nm_**a** during sequential batch cultivations with cell recycle for seven batches in LB_malt_ and **b** during sequential batch cultivations with cell recycle for four batches in MB_malt_

The production of carotenoids increased as the cell density increased in each batch (Fig. 7). In LB_malt_ carotenoid production ceased at the end of each cycle, indicating that the stationary phase was reached (Fig. 7a). A corresponding decrease was not observed in MB_malt_, in accordance with the production pattern in the single batch. The total carotenoid content in LB_malt_ increased throughout the complete sequence of batches, as shown by the absorbance at 450 nm from 0.002 at the beginning of the first batch cultivation, to 1.67 at the end of the last batch in the cultivation sequence (Fig. 8a). A similar trend was shown in the batch cultivation sequence of *R. marinus* DSM 16675 in MB_malt_ (Fig. 8b). The production of total carotenoids increased with increasing cell density in every batch, which is in accordance with the production trend observed in cultivations using LB_malt_. The produced total carotenoids in LB_malt_ were significantly higher than those in MB_malt_ (Figs. 8). To increase the production of carotenoids in MB_malt_, the cells should be kept in stationary phase for a longer time as seen in the single batch cultivation in MB_malt_ (Fig. 4b). Moreover, further study is needed to determine the concentration of total produced carotenoids to compare the total carotenoids production by *R. marinus* DSM 16675 and compare it with other thermophilic carotenoids producers.

In contrast to carotenoid production, there was poor EPSs production in LB_malt_ in each batch. The highest *Q*_E_ observed in cultivations in LB_malt_ was in batch 2 and 7, in which the cells remained in the stationary phase for a longer time period than in the other batches (Fig. 7a). The total concentration of produced EPSs was 3.77 (mg/L) with an overall *Q*_E_ of 0.1 (mg/L h) in the complete 7 batch cultivation sequence (of totally 35 h) of *R. marinus* DSM 16675 in LB_malt_.

**Fig. 9:**
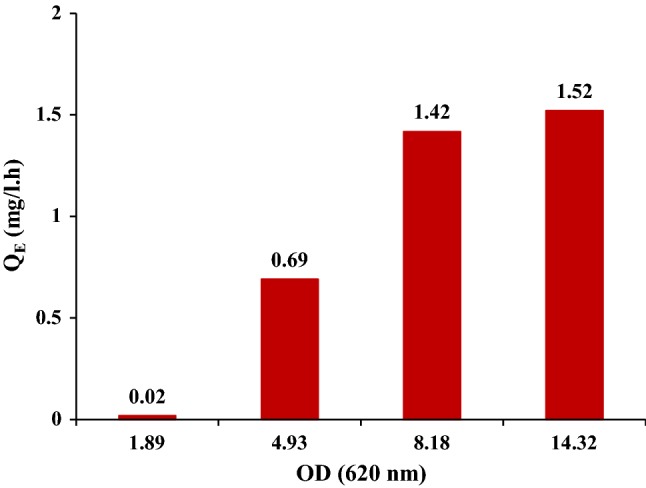
EPSs volumetric productivity (mg/L  h) of *R. marinus* DSM 16675 in each batch in cycle sequence of four batches grown in MB_malt_. Each cycle was represented by its final OD_620nm_

The EPSs production pattern in the batch sequence grown in MB_malt_ was different, as expected based on the single batch data (Fig. 3). In batch 1–3, *Q*_E_ increased significantly with growing cells, from 0.02 mg/L h in batch 1–1.42 mg/L  h in batch 3. In the last two batches (3 and 4), the productivity was stable (Fig. 9). The total production of EPSs was 19 (mg/L) with an overall *Q*_E_ of 0.67 (mg/L  h) during four batch sequences of *R. marinus* DSM 16675 in MB_malt_. Interestingly, the EPSs concentration drastically decreased between batches, meaning that most of the EPSs is in solution and not tightly bound to the cells as tightly bound EPSs. The fact that most detected EPSs, produced by *R. marinus* DSM 16675, ar excreted into the medium has not previously been reported (Fig. 7B).

The total production of EPSs by *R. marinus* DSM 16675 was low in comparison to thermophiles with good production of EPSs, shown to reach EPS concentrations in the range of 55–897 mg/L (Kambourova et al. 2016). The reason might be due to harvesting the cells at the beginning of the stationary phase, since all good EPSs producers were reported to synthesize EPSs mainly during a prolonged stationary phase at low cell densities (Kambourova et al. 2016).

## Conclusion

The present study describes different modes of operation for the cultivation of *R. marinus* DSM 16675 using two different complex media, LB_malt_ and MB_malt_. Batch, fed-batch, and sequential batch cultivation techniques were used to evaluate the cell growth and product formation, focusing on carotenoids and EPSs. The results showed that LB_malt_ was a more suitable medium than MB_malt_ to obtain high cell densities, while MB_malt_ was a better option for the production of EPSs. However, the overall EPSs productivity of *R. marinus* DSM 16675 was low (3- to 40-fold) in comparison to other thermophilic bacteria (Kambourova et al. 2016) and needs further improvement. The monosaccharide composition of the EPSs differed, as the EPSs produced in MB_malt_ also contained xylose, while this was not the case for the EPSs produced in cultures grown in LB_malt_. Carotenoids were produced by cultures grown in both media. The total carotenoids production in LB_malt_ was dependent on cell growth, while in MB_malt_, the total carotenoids production was observed in both exponential phase and stationary phase.

The key difference between the batch and fed-batch cultivations contra cell recycling (also termed sequential batch) is that the medium is exchanged between cycles, which will have removed any accumulating inhibitory substances in the medium. The presence of such substances could explain why batch and fed-batch cultivations reach stationary phase at the same cell density, even though fed-batch theoretically should reach far higher cell densities. Growth limitation due to accumulation of metabolites has previously been reported for marine microorganisms (De Carvalho and Fernandes 2010). Further work needs to be performed to identify potential metabolites causing growth limitation of *R. marinus* DSM 16675.

## Electronic supplementary material

Below is the link to the electronic supplementary material.

Supplementary file1 (DOCX 262 kb)
