# Supplementary material for: Cultivation technology development of Rhodothermus marinus DSM 16675
Source: Extremophiles. 2019 Sep 14;23(6):735–45. doi: 10.1007/s00792-019-01129-0 (PMC6801211; doi:10.1007/s00792-019-01129-0)
Supplement: Supplementary file 1 — Supplementary file1 (DOCX 262 kb) [file 792_2019_1129_MOESM1_ESM.docx]

**Fig.1** Chromatograms of HPAC analysis of monosaccharides composition of (A) hydrolyzed LB medium and (B) sugar standards (5μg/ml)

**Fig.2** Chromatograms of HPAC analysis of monosaccharides composition of (A) hydrolyzed MB medium and (B) sugar standards (5μg/ml)

**Arabinose**

**Galactose**

**Glucose**

**Mannose**

A

**Arabinose**

**Galactose**

**Glucose**

**Mannose**

**Xylose**

B

**Fig.3** Chromatograms of HPAC analysis of monosaccharides composition of produced EPSs in (A) LB_malt_ and (B) MB_malt_
